# Force-Induced Site-Specific Enzymatic Cleavage Probes Reveal That Serial Mechanical Engagement Boosts T Cell Activation

**DOI:** 10.1021/jacs.3c08137

**Published:** 2024-03-07

**Authors:** Jhordan Rogers, Rong Ma, Alexander Foote, Yuesong Hu, Khalid Salaita

**Affiliations:** † Department of Chemistry, Emory University, 1515 Dickey Drive, Atlanta, Georgia 30322, United States; ‡ Wallace H. Coulter Department of Biomedical Engineering, Georgia Institute of Technology and Emory University, Atlanta, Georgia 30332, United States

## Abstract

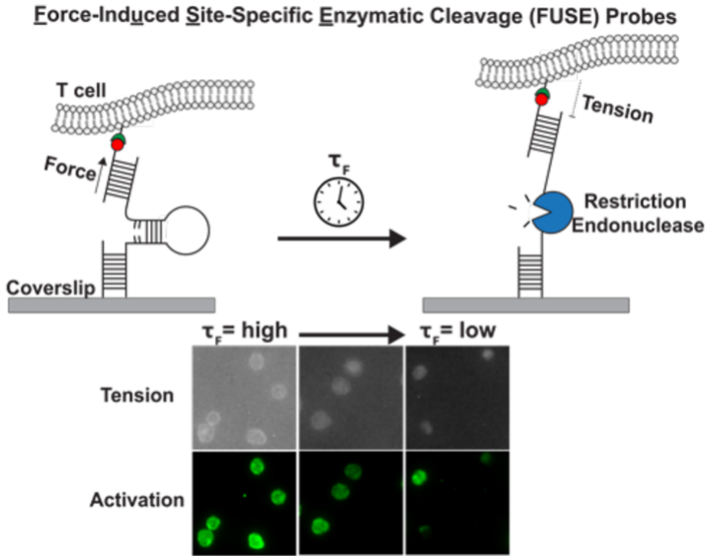

The T cell membrane is studded with >10^4^ T cell receptors (TCRs) that are used to scan target cells to identify short peptide fragments associated with viral infection or cancerous mutation. These peptides are presented as peptide-major-histocompatibility complexes (pMHCs) on the surface of virtually all nucleated cells. The TCR-pMHC complex forms at cell–cell junctions, is highly transient, and experiences mechanical forces. An important question in this area pertains to the role of the force duration in immune activation. Herein, we report the development of force probes that autonomously terminate tension within a time window following mechanical triggering. Force-induced site-specific enzymatic cleavage (FUSE) probes tune the tension duration by controlling the rate of a force-triggered endonuclease hydrolysis reaction. This new capability provides a method to study how the accumulated force duration contributes to T cell activation. We screened DNA sequences and identified FUSE probes that disrupt mechanical interactions with *F* > 7.1 piconewtons (pN) between TCRs and pMHCs. This rate of disruption, or force lifetime (τ_F_), is tunable from tens of minutes down to 1.9 min. T cells challenged with FUSE probes with *F* > 7.1 pN presenting cognate antigens showed up to a 23% decrease in markers of early activation. FUSE probes with *F* > 17.0 pN showed weaker influence on T cell triggering further showing that TCR-pMHC with *F* > 17.0 pN are less frequent compared to *F* > 7.1 pN. Taken together, FUSE probes allow a new strategy to investigate the role of force dynamics in mechanotransduction broadly and specifically suggest a model of serial mechanical engagement boosting TCR activation

## Introduction

Cytotoxic, or CD8+, T cells are essential during the adaptive immune response, as they are responsible for identifying and eradicating virally infected or cancerous cells.^1−3^ The T cell receptor (TCR) distinguishes between nonstimulatory “self” peptide and stimulatory “foreign” peptide antigens that are presented by major histocompatibility complexes (MHCs) on the surface of virtually every cell type.^4−6^ This discrimination process is extremely effective, even though there is minimal difference between the affinities of the nonstimulatory and stimulatory peptide MHCs (pMHCs), as both bind to the TCR with a *K*_D_ typically in the μM regime, which is among the weakest receptor–ligand interactions in biology.^7−12^ Additionally, T cells are ultrasensitive; CD8+ T cells can become activated by as few as one to three stimulatory pMHCs on the surface of an antigen-presenting cell.^13−16^ Although T cells are highly sensitive and specific toward aberrant cells, the molecular mechanisms that initiate their cytotoxic effector functions remain poorly understood.

**Figure 1:**
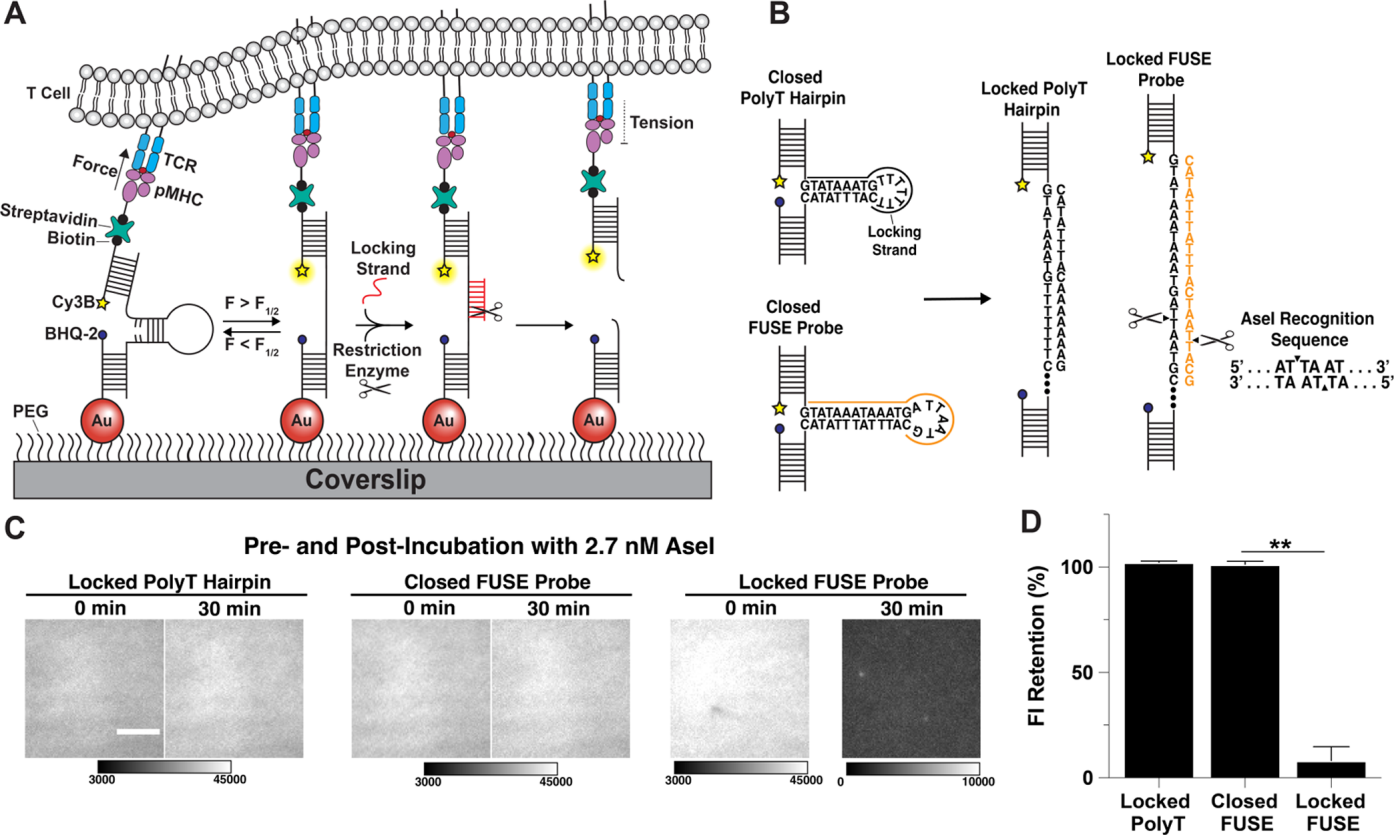
(A) Schematic of force-induced site-specific enzymatic cleavage (FUSE) assay. (B) Design of the FUSE probe compared to the traditional 4.7 pN probe with a polyT loop. The 21mer locking strand for the FUSE probe (orange) binds to the open hairpin to complete the recognition site for AseI. (C) Representative TIRF images of the locked 4.7 pN probe with polyT loop, closed FUSE probe, and locked FUSE probe before and after adding AseI restriction endonuclease. Scale bar = 5 μm. (D) Quantification of the retention of fluorescence intensity with surfaces presenting the locked 4.7 pN probe with polyT loop (102 ± 1%), the closed FUSE probe (101 ± 1%), or the locked FUSE probe (8 ± 7%) with 2.7 nM AseI. Statistical analysis performed using Student’s *t* test, **p < 0.01.

To help explain the phenomenal specificity of the TCR, one prominent model suggests that the TCR functions as a mechanosensor; mechanical forces transmitted to the TCR-pMHC complex boost its discriminatory power.^17−21^ Early single-molecule experiments showed enhanced T cell activation in response to the application of 10 pN force applied to the TCR-pMHC bond. These small, fine-tuned forces on the scale of 5–20 pN have also been suggested to stabilize the interaction between TCR and pMHC, ultimately increasing the lifetime of the bond.^22−25^ Our group provided evidence validating the mechanosensor model by developing sensors that mapped 12–19 pN T cell forces generated by the T cell cytoskeleton and transmitted to their TCR-antigen bonds during antigen recognition.^26−58^ Briefly, molecular force probes visualize TCR forces by presenting pMHC ligands conjugated to fluorescently labeled DNA hairpins that are immobilized onto a glass coverslip (Figure S1). These probes contain a fluorophore–quencher pair attached to the termini of the hairpin stem. Once a TCR binds to the antigen and exerts *F* greater than the *F*_1/2_ of the hairpin (50% probability of unfolding the hairpin at equilibrium), then the fluorophore is separated from the quencher, leading to a 100-fold increase in fluorescence intensity (Figure 1A).

Another prominent model that helps explain TCR sensitivity is the serial engagement model which postulates that TCRs repeatedly engage stimulatory pMHCs to trigger activation at low antigen density.^29−34^ Kalergis et al. have demonstrated a “Goldilocks-like” relationship between the TCR-pMHC dwell time and T cell activation.^9^ If dwell times are too short, then the TCRs fail to initiate signaling. However, if dwell times are too long, then few TCRs benefit from consistent stimulation, and T cell activation may be dampened.^35,36^ Despite the accumulating experimental support for both the serial engagement model and the mechanosensor model, it remains unclear how these models work together.

Accordingly, the primary goal of this work is to develop a tool to explore how these two models may operate together to enhance TCR triggering. Here, we introduce force-induced site-specific enzymatic cleavage (FUSE) probes, which allow one to tune the duration of the TCR-pMHC force (Figure 1A). FUSE probes contain the core components of DNA hairpin molecular force probes but are degraded after the antigen is mechanically sampled. This process occurs by adding a single-stranded DNA (locking strand) that is complementary to the cryptic loop region of the hairpin that is only exposed once the probe is unfolded.^27^ This cryptic region contains a sequence that is recognized by a site-specific restriction endonuclease only when the locking strand hybridizes with the unfolded probe. This selective cleavage disrupts the mechanical resistance of pMHC, as it is no longer tethered to the surface by a DNA duplex. The rate of this disruption is tuned by varying the concentration of nuclease added, which allows the duration of mechanical resistance or the tension duration experienced by TCRs, to be controlled orthogonally and without modification to the antigen or TCR.

We demonstrate that the cleavage of FUSE probes with the AseI restriction endonuclease is highly site-specific. Surface rate measurements of the locking strand binding to FUSE probes, as well as the rate of enzymatic cleavage of the locked probe, showed that FUSE probe cleavage is highly selective, and closed probes are cleaved at a rate 10^3^ lower than that of opened probes. Force lifetimes following mechanical triggering are tunable down to 1.9 min, which was achieved at 10.7 nM AseI. FUSE probes presenting a pMHC loaded with the ovalbumin-derived peptide, SIINFEKL, were used to demonstrate that FUSE probe cleavage was unperturbed at the junction between a T cell and antigen-coated substrate. Critically, antigen-FUSE probes terminate tension but remain confined at the T cell surface because of rebinding to high-density TCRs as we measured a half-life of 12.8 min for released probes. Lastly, early T cell signaling, as reflected by the ZAP70 phosphorylation level at *t* = 15 min, is dampened with 7.1 pN FUSE probes, and this dampening is further enhanced at low antigen density. These results lead to the conclusion that serial mechanical engagement boosts T cell activation.

## Results

### Characterization of FUSE Probes

FUSE probes differ from traditional hairpin probes in their force-triggered self-cleavage response. We aimed to achieve this function by incorporating a recognition sequence for a site-specific restriction endonuclease in the loop segment of the DNA hairpin that can be cleaved only upon mechanical melting of the hairpin (Figure 1B). Specifically, the endonuclease recognition sequence is exposed only once a complementary “locking strand” hybridizes to the mechanically unfolded probe. Thus, the locking strand must exhibit two features: the first is rapid and specific hybridization to unfolded FUSE probes. The second is that the “locked probe” must demonstrate high thermostability at 37 °C for optimal enzymatic activity (Figure S2A). To meet these criteria, we designed and screened a small library of FUSE sequences (Table S1 and Figure S2B).^31,32^ Our initial sequences were unsuccessful as we found that FUSE probes incorporating our previously reported 4.7 pN probe design (stem = 22% GC, 9 bp) resulted in poor thermostability at 37 °C once it was locked with its accompanying 15-nt locking strand (Figure S2C).^27^ Ultimately, we found that extending the stem of the hairpin to 13 bp, replacing the seventh T base in the loop with a G, and increasing the locking strand length to 21-nt, led to increasing the stability of the locked probe substantially. The optimized sequence is shown in Figure 1B, and the *F*_1/2_ of this sequence was 7.1 pN as calculated using established precedent (eqs S1 and S2).^28,37,38^ Since site-specific enzymatic cleavage is imperative for specifically perturbing mechanical interactions, we next aimed to quantify AseI activity and specificity. Here, we introduced AseI to a surface-tethered locked 4.7 pN probe with a polyT loop, closed 7.1 pN FUSE probe, and locked FUSE probe.^26,27^ Note that the locked probes were labeled with a Cy3B-BHQ-2 fluorophore–quencher pair, while the closed 7.1 pN FUSE probe included only the Cy3B reporter to facilitate quantifying cleavage rates. By measuring the time-dependent decay of the Cy3B signal associated with the top strand of each probe, we were able to determine the specificity and activity of AseI on a surface-tethered substrate. Importantly, under our conditions, there was no observable cleavage of the locked nonspecific hairpin probe with a polyT loop that was lacking the recognition sequence. The closed FUSE probe, which only contains the incomplete recognition sequence, also did not show any detectable cleavage (Figure 1C). In contrast, 90% of the locked FUSE hairpin was cleaved under identical conditions after a 30 min incubation at 37 °C with 2.7 nM AseI (Figure 1C,D).

**Figure 2:**
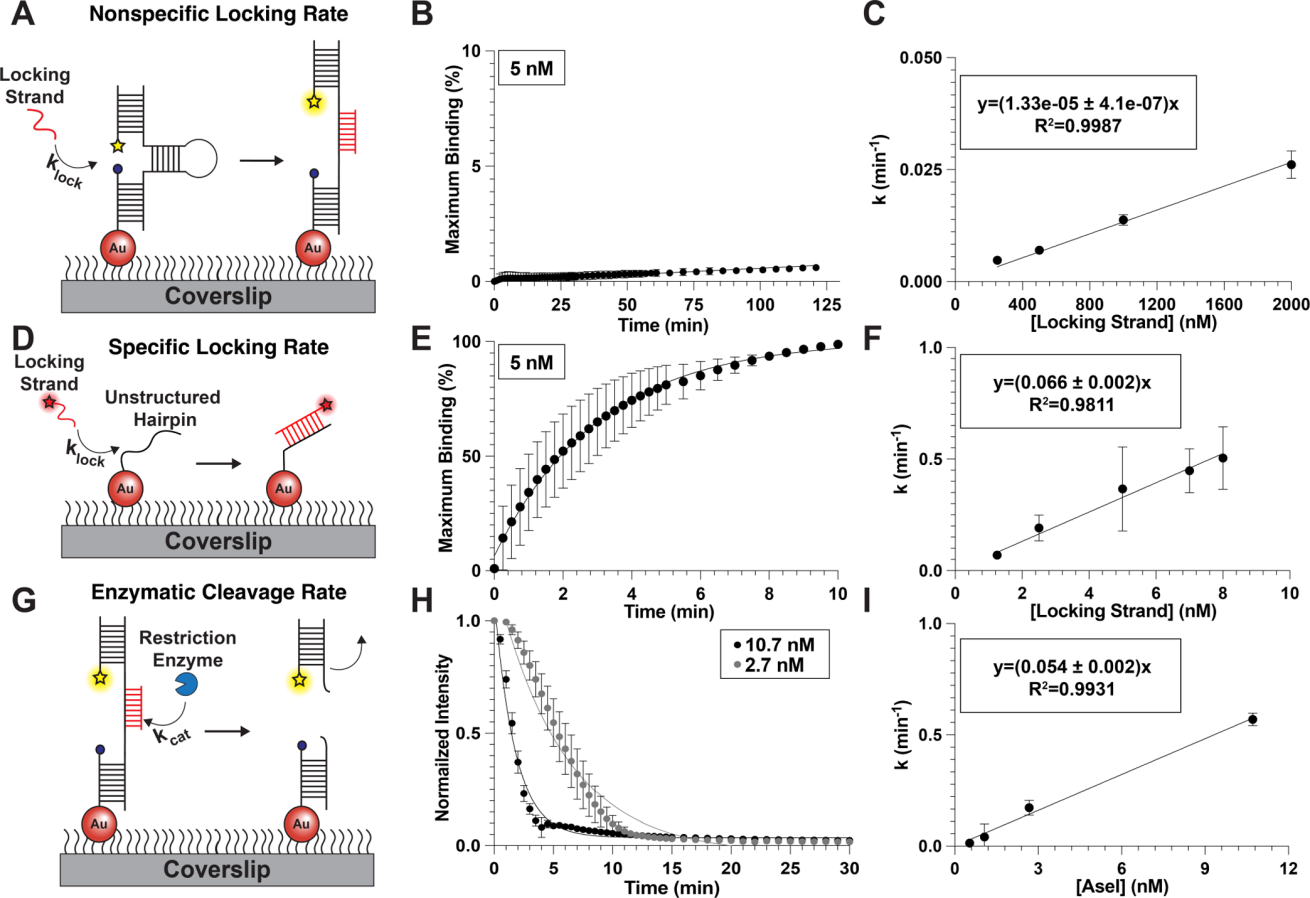
(A) Schematic of the experiment to quantify the rate of nonspecific hybridization of the locking strand. (B) Representative plot of the percentage of maximum locking strand binding to the hairpin versus time to quantify the nonspecific rate of hybridization for 5 nM of locking strand, *k* = 5.75 × 10^–5^ ± 3.4 × 10^–6^ min^–1^. (C) Plot of the pseudo-first-order rate constant of nonspecific hybridization versus concentration of locking strand. (D) Schematic of experimental design to quantify the rate of specific hybridization of locking strand to its exposed docking site. (E) Representative plot of the percentage of maximum locking strand binding to the unstructured hairpin versus time to quantify the specific rate of hybridization for 5 nM of locking strand, *k* = 0.366 ± 0.189 min^–1^. (F) Plot of the pseudo-first-order rate constant for specific hybridization versus the concentration of locking strand. (G) Schematic displaying the experimental setup to quantify the rate of enzymatic cleavage of a locked hairpin. (H) Representative plot of the normalized surface intensity versus time to determine the rate of cleavage and τ_F_ for 2.7 and 10.7 nM of AseI. The apparent rate of cleavage for 2.7 nM AseI was 0.173 ± 0.0.034 min^–1^ and 0.570 ± 0.0.029 min^–1^ for 10.7 nM. (I) Plot of the apparent rate of cleavage versus concentration of AseI.

Next, we aimed to quantify the kinetics of FUSE triggering. This includes nonspecific hybridization between a closed FUSE probe and its locking strand, specific hybridization between an unfolded FUSE hairpin and its locking strand, and the rate of enzymatic cleavage of the locked probe (Figures 2 and S3). To determine the lock hybridization kinetics, we coated coverslips with either the closed FUSE probe or the “unstructured hairpin”, which is a single-stranded DNA that only contains the portion of the hairpin that is complementary to the locking strand. We then added either unlabeled or fluorophore-labeled locking strand and observed the increase in the signal associated with the hairpin opening or locking strand binding to the unstructured hairpin (Figure 2A,D). The increase in this signal can be fitted to a one-phase association curve (eq S3), which allows the apparent rate, k, to be determined for a given concentration of locking strand (Figures 2B,E and S2B,D). We then plotted the rate versus locking strand concentration, which allowed us to extrapolate a rate for any given locking strand concentration, assuming pseudo-first-order kinetics (Figure 2C,F). To quantify the rate of enzymatic cleavage of the locked probe, the FUSE probe was annealed with its locking strand prior to conjugation to the surface (Figure 2G). Then, AseI restriction endonuclease concentrations ranging from 0.5 to 10.7 nM were incubated at 37 °C for 30 min (Figure S3F). The enzymatic activity of AseI was observed by tracking the decrease in signal as the fluorophore-labeled top strand diffuses away from the surface after cleavage. This decay was fit to a one-phase exponential decay curve (eq S4) to determine the half-life (*t*_1/2_) and the average lifetime of the locked probe (τ_F_) (Figures 2H and S3F). We then plotted this rate of decay versus AseI concentration to compare enzymatic activity across a range of endonuclease concentrations (Figure 2I).

For FUSE probes to terminate mechanical signaling accurately and rapidly, the specific hybridization and enzymatic cleavage rates must be much faster than the rates of nonspecific hybridization and cleavage. Indeed, we report a 5 × 10^3^ faster specific hybridization rate (328 ± 11 min^–1^) compared to the nonspecific hybridization rate (0.066 ± 0.006 min^–1^) for 5 μM locking strand, which is the concentration we chose for the FUSE assay (Figure 2B,C,E,F). While this quantification for locking strand hybridization is performed at *F* = 0, we modeled the stability of locked FUSE probes for a range of locking strand concentrations as *F* increases from 0 to 20 pN (Figure S4).^39^ We found that locked FUSE probes are resilient to forces up to 20 pN at our working concentration of 5 μM locking strand (Figure S4B,C). This suggests that any changes in the off-rate associated with receptor-mediated forces do not outweigh the overwhelmingly high on-rate associating with the concentration of locking strand used for our FUSE assay. Additionally, we found that the τ_F_ of the locked probe was 5.8 ± 0.7 min at 2.7 nM and 1.9 ± 0.3 min at 10.7 nM AseI, and this average lifetime does not change significantly as we decreased the incubated probe concentration by as low as 20-fold (Figure S5). These values suggest that FUSE probes are able to be effectively locked and cleaved upon mechanical triggering.

### FUSE Probes Can Dynamically Map and Disrupt TCR–Ligand Mechanical Interactions

**Figure 3:**
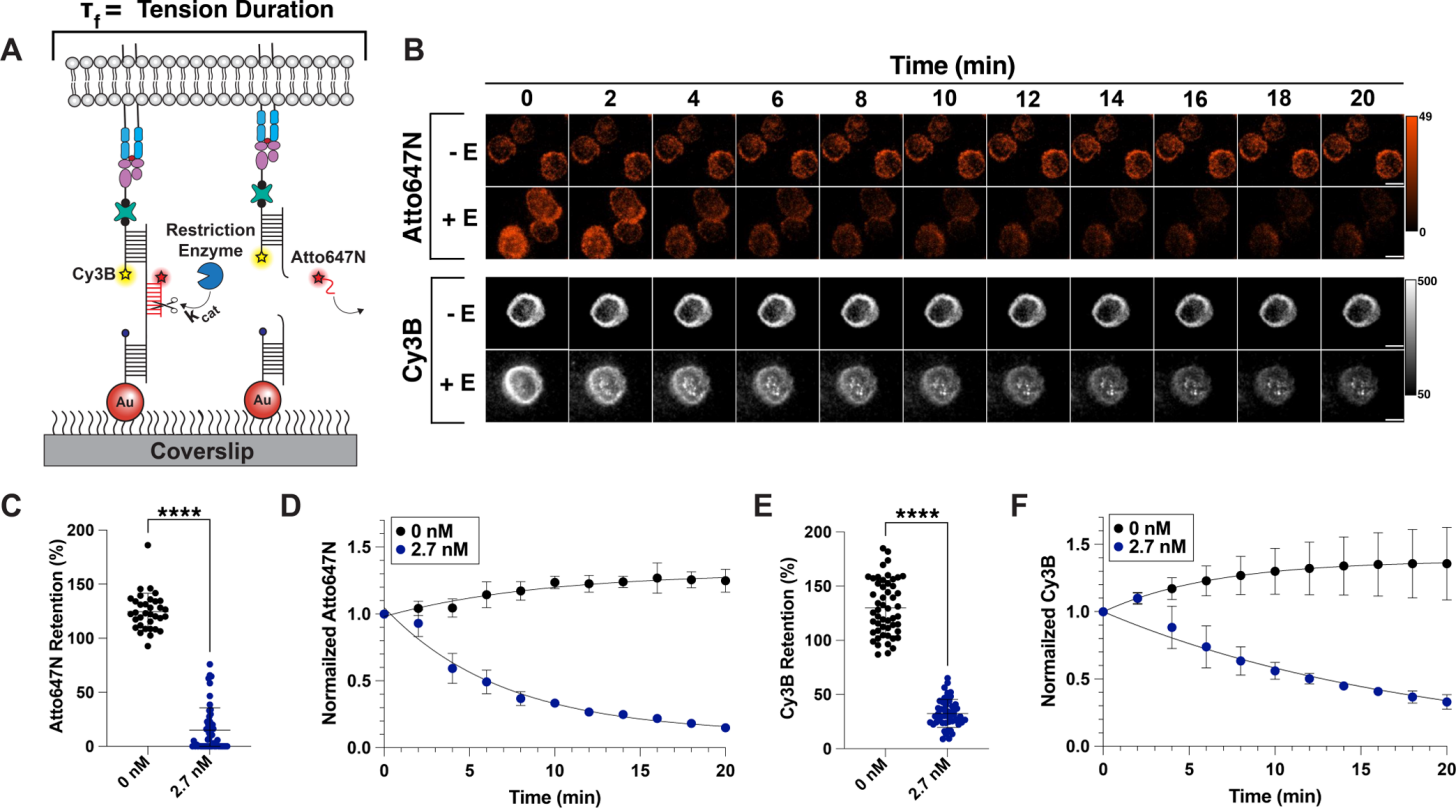
(A) Schematic showing the cleavage of locked FUSE probes under cells; the rate of this enzymatic cleavage defines the average tension duration experienced by TCRs. (B) Representative timelapse showing the decay of locked FUSE probes under cells. Atto647N signal tracks the locking strand, while Cy3B signal is used to visualize the ligand and top strand of the FUSE probe. Scale bar = 5 μm. (C) Quantification of the change in locking strand signal under cells after incubation with or without AseI enzyme, *n* > 30 cells for each condition. Statistical analysis was performed using Student’s *t* test, ****p < 0.0001. (D) Plot showing the change in lock signal under cells over the course of a 20 min incubation with (blue) or without (black) AseI. (E) Quantification of the depletion of ligand under cells after incubation with or without AseI, *n* > 30 cells for each condition. Statistical analysis was performed using Student’s *t* test, ****p < 0.0001. (F) Plot showing the exponential decay of ligand under cells over the span of 20 min in the presence of AseI (blue) and a slight increase in tension signal in the absence of AseI (black).

After validating the kinetic parameters of these probes with cell-free experiments, we next wanted to ensure that FUSE probes can be employed to study receptor–ligand interactions. Although the prior cell-free experiments demonstrated the feasibility of this assay, it was unclear whether the cellular environment would impede the enzyme’s ability to access the locked substrate. For these experiments, we elected to use the well-studied OT-1 transgenic T cell model, in which TCRs are reactive to the ovalbumin SIINFEKL peptide (N4). We decorated 7.1 pN FUSE probes with N4 pMHC ligand.^7,25−27,40^ The *F*_1/2_ of 7.1 pN was chosen because this force magnitude is well within the range that OT-1 TCRs transmit to the N4 pMHCs.^25,27^ Briefly, we allowed the OT-1 cells to spread on coverslips for 10–15 min with either fluorophore-labeled or unlabeled locking strand to allow the locked tension signal to accumulate (Figure S6). We then added or withheld AseI and tracked the signal of either the fluorophore associated with the locking strand (Atto647N), or the fluorophore associated with the top strand/pMHC (Cy3B) underneath cells (Figure 3A,B). In Figure 3B, both the Atto647N and Cy3B signals are associated with tension; however, the decay of the Atto647N signal reports the rate of enzymatic cleavage of the locked probe. Since the Cy3B dye is conjugated to the antigen strand, its decay is hindered due to rebinding to TCRs that may occur after the probe is released from the surface. Indeed, the rate of decay of the lock signal (Atto647N) is robust, with only 15% of the initial signal remaining under cells after AseI is added (Figure 3C). While this depletion is apparent, this decay happens at a slightly slower rate compared with the cell-free cleavage assay (τ = 6.9 min) (Figure 3D). In contrast, the fluorophore associated with the ligand (Cy3B) depletes at a much slower rate (τ = 18.5 min), and around 40% of the initial signal remains after a 20 min incubation with AseI (Figure 3E,F). We speculate that this slower rate of Cy3B depletion is due to TCRs rebinding pMHCs once these probes are cleaved from the surface. Altogether, these results show that FUSE probes are effectively cleaved at the T cell–substrate junction and demonstrate extended dwell time at the T cell surface despite termination of mechanical resistance.

### TCR-pMHC Tension Duration Influences T Cell Activation

**Figure 4:**
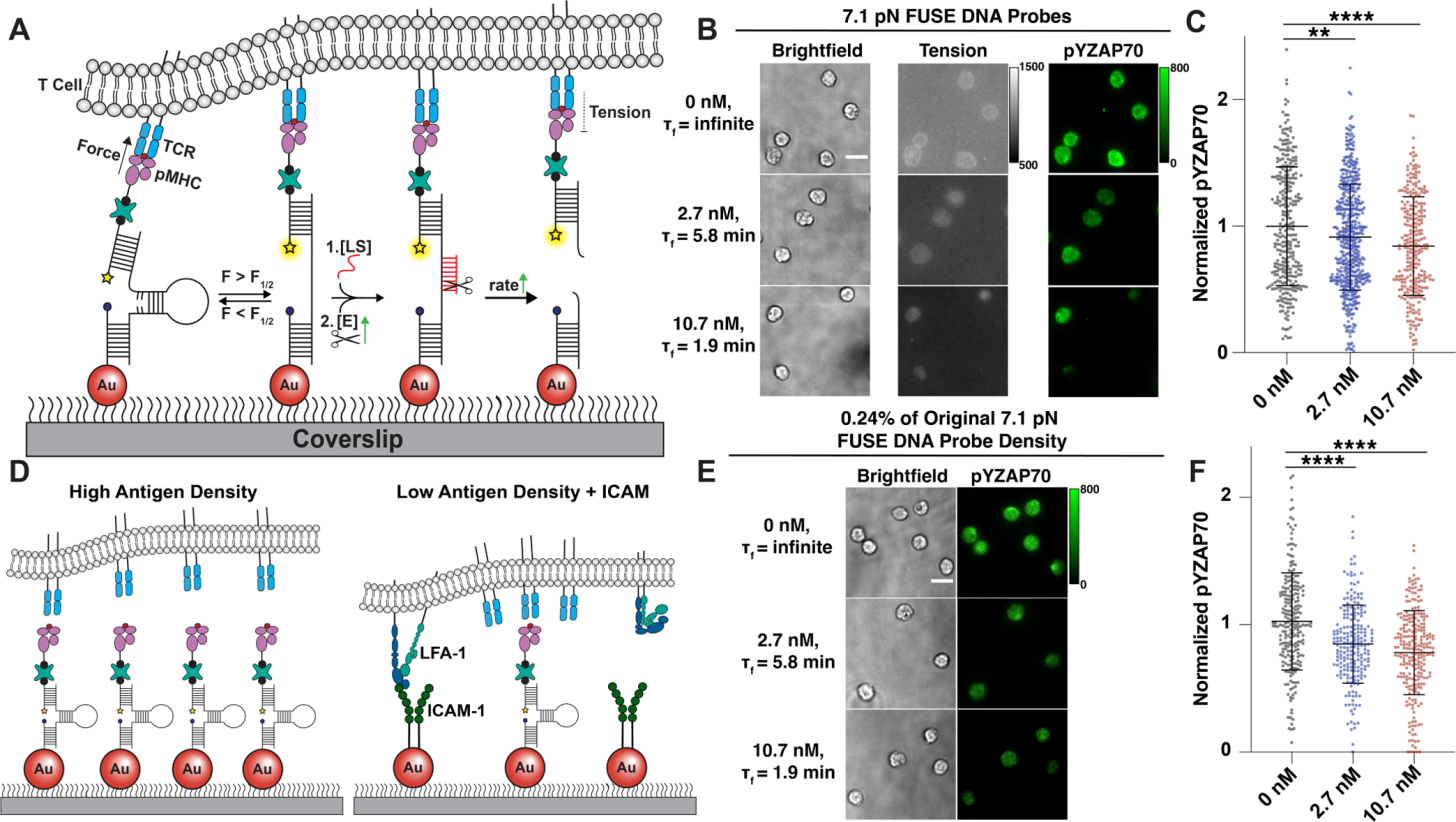
(A) Schematic depicting the decrease in TCR-pMHC τ_F_ when restriction endonuclease concentration is increased on surfaces presenting FUSE probes. (B) Representative images showing the difference in tension and pYZAP70 signal after a 15 min FUSE assay with three different concentrations of enzyme (0, 2.7, 10.7 nM). Scale bar = 10 μm. (C) Quantification of pYZAP70 signal after OT-1 cells were incubated with 0 nM (mean norm. pY = 1.00), 2.7 nM (mean norm. pY = 0.92), and 10.7 nM (mean norm. pY = 0.85) of AseI on FUSE probes, *n* > 300 cells from three biological replicates. Statistical analysis was performed using Student’s *t* test between no enzyme control and experimental groups, **p < 0.01 and ****p < 0.0001. (D) Schematic depicting cell interactions on a high-density versus a low-antigen-density surface. (E) Representative images showing the difference in pYZAP70 signal after a 15 min FUSE assay without AseI enzyme, with 2.7 nM AseI, and with 10.7 nM AseI. Scale bar = 10 μm. (F) Quantification of pYZAP70 signal in cells after incubation without enzyme (mean norm. pY = 1.02), with 2.7 nM enzyme (mean norm. pY = 0.85), and with 10.7 nM AseI (mean norm. pY = 0.78), *n* > 200 cells from three biological replicates. Statistical analysis was performed using Student’s *t* test, ****p < 0.0001.

After demonstrating that FUSE probes are triggered and selectively cleaved in response to *F*> 7.1 pN, we aimed to test the role of the accumulated TCR-pMHC tension duration on T cell signaling. Specifically, we were interested in quantifying early T cell activation in response to TCR-pMHC tension duration. To achieve this, we measured phosphorylation of the proximal kinase ZAP70 (pYZAP70) in OT-1 cells interacting with N4 antigen presented by FUSE probes. By titrating enzyme concentration, we were able to vary the average length of time that TCRs can mechanically interact with surface-bound pMHCs, otherwise known as τ_F_ (Figure 4A). In other words, greater concentrations of AseI will diminish the duration of time that an antigen offers mechanical resistance to TCR forces. By decreasing τ_F_ and measuring the activation status of interacting cells, we can infer a causal relationship between repeated and durable mechanical receptor–ligand interactions and T cell activation. If full TCR triggering is achieved with single, short-lived mechanical interactions, then we would see no difference in activation as a function of τ_F_. We established the baseline, “maximum τ_F_” as the condition when no enzyme was added. This control group provided the pYZAP70 baseline level that we compared against conditions where either 10.7 nM (τ_F_ = 1.9 min) or 2.7 nM (τ_F_ = 5.8 min) AseI was added to cells along with 5 μM locking strand. Interestingly, we observed a 15% decrease in pYZAP70 expression when the τ_F_ was reduced to 1.9 min, but only an 8% decrease when τ_F_ was reduced to 5.8 min (Figure 4B,C). Figure 4B also shows a decrease in tension signal as enzyme concentration increases due to the increased rate of cleavage of locked FUSE probes that corresponds with a decrease in τ_F_. Additionally, we limited the τ_F_ of FUSE probes presenting antiCD3ε to 5.8 min and observed a more pronounced reduction in pYZAP70 (15%) compared to probes presenting pMHC with the same τ_F_ (8%) (Figure S7). We hypothesize that this larger decrease in the antiCD3ε condition was due to the disruption of long-lived mechanical interactions between antiCD3ε and the TCR, as antiCD3ε displays higher affinity (nM *K*_D_) toward the TCR than pMHCs (μM *K*_D_).^41,42^ Note that both the AseI hairpin and the accompanying locking strand must be present to observe a statistically significant decrease in T cell signaling, thus validating that selective FUSE probe cleavage is responsible for this perturbation (Figure S8).

Since antigen is depleted from the surface after mechanical interaction, we aimed to validate that the decrease in early T cell activation relied on changes in the τ_F_—not only a decrease in antigen density on the surface. By decreasing the concentration of the FUSE probe incubated on the surface by half, we demonstrated that pYZAP70 levels were unaffected by a 60% reduction in antigen density (Figure S9). These results, along with our previous results (Figure 3F) showing that >40% of antigen remains underneath cells 15 min after cell engagement, indicate that potential fluctuations in antigen availability caused by FUSE are not responsible for changes in T cell activation shown in Figure 4. Thus, we conclude that perturbation in T cell signaling is dictated by the length of tension duration allowed by FUSE probes, which ultimately governs the number of mechanical engagements TCRs experience.

To further validate this conclusion, we investigated the impact of decreasing the probe density down to single-molecule antigen density, as well as increasing the trigger force threshold of FUSE probes. Since serial engagement should be more pronounced at low antigen density, we hypothesized that decreasing τ_F_ would also have greater impact on T cell activation at low ligand density.^9^ Therefore, limiting the τ_F_ of cells interacting with a vastly decreased antigen density should result in a greater reduction in the activation potential. Thus, we incubated 40,000 times less FUSE probe (5 pM instead of 200 nM) to achieve single-molecule probe density, which resulted in a 400-fold depletion of antigen on the surface compared to our initial assay (Figures S10 and S11, Movie S1). Since T cells fail to spread on surfaces presenting low levels of antigen, we also anchored dimeric ICAM-1 along with pMHC-FUSE probes to promote adhesion (Figures 4D and S12). Using these low-antigen-density ICAM surfaces, we examined pYZAP70 expression in cells after a 15 min incubation with our FUSE probes. Interestingly, we observed a decrease in pYZAP70 of 15% when τ_F_ was limited to 5.8 min and 23% when τ_F_ was limited to 1.9 min on surfaces presenting a single-molecule density of FUSE probes, which was a more pronounced difference than what was observed in the initial “high” antigen density experiments (Figures 4E,F and S13). We also found that TCRs formed dense clusters in regions corresponding with tension signal on surfaces presenting low antigen density, whereas T cells interacting with surfaces presenting a high density of antigen organized their TCRs more homogeneously through the cell–substrate interaction (Figure S14). We then visualized repetitive mechanical sampling on low-antigen-density surfaces using the 4.7 pN short-stem FUSE probe and a 15-nt locking strand with low thermostability (Figure S15, Movies S2 and S3). By using a FUSE probe-locking strand system with decreased thermostability, we were able to keep mechanically sampled probes open long enough to be imaged without locking them in the open conformation permanently (Figure S15B–D). Altogether, these findings suggest that the effects of serial mechanical engagement are exaggerated at lower antigen densities, which is consistent with models that describe how serial engagement may contribute to T cell stimulation at a very low antigen density.

Next, we created a FUSE probe with an *F*_1/2_ of 17.0 pN to test signaling in cells interacting with probes that are less likely to be unfolded and cleaved. We have previously shown that OT-1 cells can easily unfold hairpins presenting N4 pMHC with an *F*_1/2_ of 12 but not 19 pN.^21^ Therefore, we were expecting a background-level signal associated with the 17.0 pN FUSE probe unfolding and thus very little change in activation associated with a decrease in the τ_F_ of TCR-pMHC interactions greater than 17 pN. However, we were able to detect tension signal using the locking strategy, albeit much lower than the tension signal reported with the 7.1 pN probe (Figure S16). When τ_F_ was limited to 1.9 min using high density of the 17.0 pN probe, we observed a 12% decrease in pYZAP70 with a lower statistical significance than the same experiment with the 7.1 pN FUSE probe (*p* < 0.05 for 17 pN, *p* < 0.0001 for 7.1 pN) (Figure S17).

## Discussion

Previous work has generated evidence supporting the TCR mechanosensor and serial engagement models as mechanisms for TCR triggering. Interestingly, the serial engagement model is primarily understood from the perspective of a series of connected reactions that are under kinetic control such that repeated binding of an antigen by different TCRs in proximity enhances and augments activation to allow for ultrahigh sensitivity.^4,43,44^ The mechanisms mediating the mechanosensor model are proposed to include kinetic mechanisms, such as catch bonds, as well as structural mechanisms where forces expose cryptic binding sites to the antigen.^17,45−48^ These models are not necessarily mutually exclusive, and as such, it is plausible that both models contribute to the sensitivity and specificity of the TCR. That said, there is little experimental evidence supporting the notion that the TCR takes advantage of both processes simultaneously, which we describe as serial mechanical engagement.

**Figure 5:**
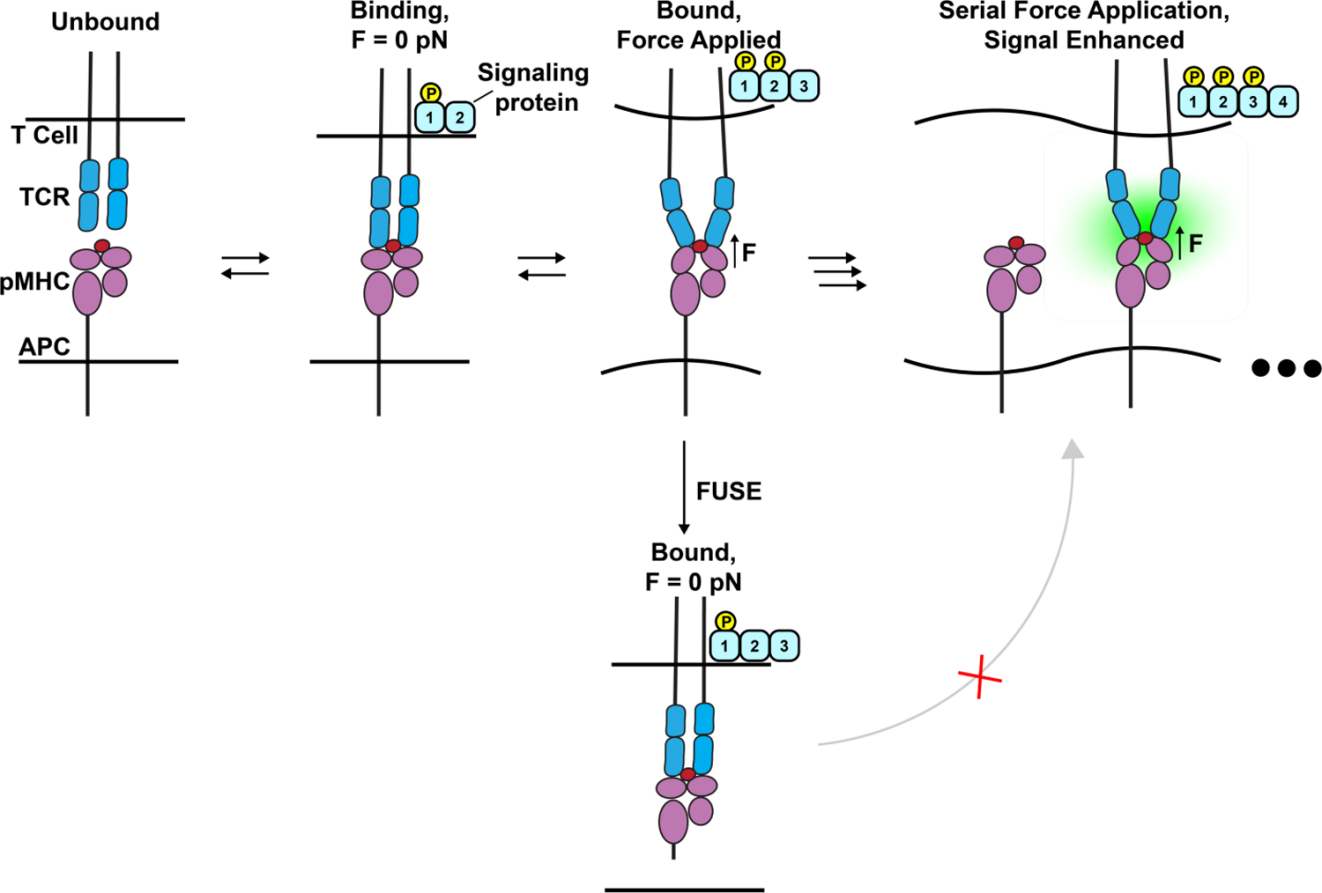
Schematic summarizing the correlation between T cell activation and the maximum TCR-pMHC tension duration afforded by the FUSE probes.

Our FUSE method provides a unique way to test the contributions of serial mechanical engagement, as our probes provide the experimenter with control over the length of time an antigen remains tethered to a surface once it is mechanically sampled with a precise pN tug. In Figure 5, we illustrate an example of the serial engagement model by showing the progression of TCR stimulation as mechanical sampling occurs on cognate antigens. FUSE probes are able to prematurely terminate this mechanotransduction, which likely prevents TCRs from continuously achieving any force-driven conformational changes or catch bond formation to promote T cell activation.^49,50^ This design differs from probes that instantaneously rupture in response to force, such as DNA tension gauge tethers (TGTs), as these probes rupture and disrupt forces as soon as force is exerted onto an antigen.^51^ Experiments using the TGT binary response to force disruption provide evidence for the mechanosensor model but cannot explore the contributions of serial engagement on mechanosensing. T cells plated on 12 pN OVA TGTs displayed a decrease of ∼60% in proximal kinase signaling compared to cells plated on 56 pN TGTs that cannot be opened by TCR forces.^26^ By definition, the TGT threshold is estimated at 2 s, and hence, the 60% dampening in pYZAP70 levels for the 12 pN TGT represents the upper limit of how force duration reduces activation. This prior literature can be compared to our findings, wherein τ_F_’s of 1.9 and 5.8 min led to a decrease in mean pYZAP70 of 15% and 8%, respectively, compared to an infinite τ_F_ probe. These findings demonstrate that the length of time that TCRs can mechanically interact with their antigens or the number of successful mechanical interactions dictates the level of activation achieved by the T cell. Importantly, our controls showing the change in ligand density on the surface and the length of time that ligands persist under cells after cleavage further corroborate the conclusion that tuning the duration of serial mechanical engagement is responsible for the change in T cell signaling observed.

Another key finding from this work is the exaggerated depletion of pYZAP70 that is observed when τ_F_ is decreased on surfaces presenting low antigen density. This experiment most accurately depicts the physiological T cell antigen-presenting cell interface, as T cells encounter remarkably few stimulatory pMHCs on the surface of antigen-presenting cells as they initiate their cytotoxic functions. Previous work has suggested that the reliance on serial engagement for TCR triggering is most pronounced when few agonist pMHCs are available to interact with a T cell.^9^ This premise is mirrored in our results, as the reduction in pYZAP70 was exaggerated after lowering the τ_F_ on surfaces presenting ∼1 antigen/μm^2^. Additionally, we found that limiting the τ_F_ of *F* > 17.0 pN, which approaches the maximum magnitude TCR-pMHC force, resulted in a 12% decrease in pYZAP70 levels with τ_F_ reduced to 1.9 min (*p* < 0.05). The reduced impact on proximal kinase activity is most likely because interactions inducing a higher magnitude of force are less frequent, and thus fewer FUSE probes are mechanically triggered and cleaved. Therefore, serial mechanical engagement is likely operating across a range of forces but with a lower frequency at *F* > 17.0 pN.

The relationship between the τ_F_ of the TCR-pMHC interaction and T cell activation potential illustrated by FUSE is well supported by previous work using single-cell force spectroscopy.^25,52^ While it has been well established that TCR-pMHC bond kinetics under force dictates the T cell’s functional response,^17,22,25,53,54^ Pryshchep et al. demonstrate that the duration of cyclic, serially applied force onto TCRs also influences the activation potential of the cell.^52^ Here, they demonstrate that intracellular calcium levels are ∼20% lower in T cells that have encountered antigens serially applying forces for ∼2 min compared to calcium levels observed after 10 min of cyclic antigen engagement. Interestingly, little flux in calcium was observed when no forces were applied during a 10 min interaction between an antigen-decorated red blood cell and a T cell. While FUSE probes limit the duration of serial TCR-mediated forces instead of ligand-mediated forces, limiting this duration reduced T cell activation by 15–23% depending on the ligand density, which agrees very well with the 20% reduction indicated in previous work. Our work further illustrates the necessity of serial mechanical engagements between TCRs and pMHCs, as the serial application of intrinsic (T cell-generated force) and extrinsic forces (applied by the experimenter) both lead to an increased T cell response over time. Moreover, the TCR-pMHC complex studied here has a bond lifetime ranging from ∼0.1 to 1 s, and thus our findings tuning force duration to 1.9 min demonstrates the importance of the integral of accumulated mechanical events rather than the outcome of single mechanical encounters in tuning T cell activation.^25^ This conclusion is in line with the notion of accumulated catch bonds triggering T cell activation as described by Zhu, Evavold, and colleagues.^25,52^

Ultimately, the development of FUSE probes has generated evidence of a link between two of the most prominent models used to explain T cell activation. In addition to this discovery, FUSE probes have a wide range of applicability toward other mechanosensitive receptors, such as integrins, Notch, and cadherins.^58,55−57^ In this work, we have characterized these probes, and now we would like to highlight some critical design parameters that one must consider before implementing this assay to study other receptors of interest. The core components of FUSE probes consist of a DNA hairpin, locking strand, and site-specific restriction endonuclease. All of these elements can be customized depending on the anticipated force range of the receptor–ligand interaction and the desired kinetics for disrupting this interaction. It is important to note that the FUSE probe must be thermodynamically stable in the closed conformation when no force is applied, and the hairpin-locking strand duplex must be thermodynamically stable (low *k*_off_) after hairpin opening. Locking and enzymatic cleavage must be rapid and specific to minimize the background antigen depletion. Note that both of these processes can be perturbed at high force magnitudes, so it is necessary to validate locking and enzymatic cleavage of FUSE probes experiencing >20 pN.^39,59^

While this design was able to investigate the connection between mechanosensing and serial engagement, optimization of FUSE could lead to further elucidation of the mechanisms of T cell activation. One limitation of the current design is that the lowest τ_F_ that we were able to achieve was still in the minute time regime, while TCR-pMHC interactions occur in the second to subsecond regime. To enhance this rate of disruption, future generations of FUSE could include a locking strand-restriction endonuclease conjugate that should ensure rapid enzymatic cleavage once the locking strand binds to the open hairpin. Another future direction for this project would be to use supported lipid bilayers to anchor FUSE probes instead of a glass substrate. This will allow antigens to freely diffuse on the surface, which mimics their physiological mobility on the surface of antigen-presenting cells. As previously mentioned, FUSE could also be used to study the effects of tension duration on any mechanosensitive receptor of interest such as Notch, cadherins, and integrins. In fact, the highly tunable design of this probe provides a sturdy framework for multiple FUSE probes with different recognition sequences to be used to study the role that tension duration plays in a variety of receptors and coreceptors simultaneously.

## Supporting Information Available

Materials and methods, list of oligonucleotide sequences, parameters for locking strand stability simulation, complete kinetic characterization of FUSE, representative images and analysis of tension and pYZAP70 signal, validation of single-molecule imaging, analysis of tension signal-TCR colocalization, and additional details (PDF)
Single-molecule probe density (Movie S1) (AVI)
Side-by-side view of single-molecule tension (Movie S2) (AVI)
Merged single-molecule tension (Movie S3) (AVI)

## Supplementary Material

ja3c08137_si_001.pdf

ja3c08137_si_002.avi

ja3c08137_si_003.avi

ja3c08137_si_004.avi

## Abbreviations

TCR: T cell receptor
pMHC: peptide-major histocompatibility complex
FUSE: force-induced site-specific enzymatic cleavage
ZAP70: zeta-chain-associated protein kinase 70
N4: ovalbumin-derived SIINFEKL peptide antigen
ICAM-1: intercellular adhesion molecule-1
TGT: tension gauge tether
